# Enhanced Recovery After Surgery (ERAS) in Renal Transplantation Patients

**DOI:** 10.4274/TJAR.2024.241702

**Published:** 2025-02-11

**Authors:** Pelin Karaaslan, Tümay Uludağ Yanaral

**Affiliations:** 1 İstanbul Medipol University Faculty of Medicine Department of Anaesthesiology and Reanimation, İstanbul, Türkiye

**Keywords:** Anaesthesia, cost-effectiveness, ERAS, quality, renal transplantation

## Abstract

Enhanced recovery after surgery (ERAS) is a set of methods that provide early recovery with a multimodal approach in the perioperative care pathway. ERAS protocols are widely used worldwide in major surgery to improve surgical outcomes and require multidisciplinary collaboration. Using ERAS protocols means better pain management, earlier mobilization, improved oral nutrition, shorter hospital stays, and cost-effectiveness. ERAS protocols were introduced into renal transplant programs quite late. Transplantation patients are challenging and should be well prepared and followed up for ERAS. This review article highlights preoperative, intraoperative, and postoperative important points for preparing patients for renal transplantation for early recovery.

**Main Points**

• Enhanced recovery after surgery (ERAS) protocols in renal transplantation by adapting them to the specific dynamics of the transplantation process.

• The ERAS program should be considered a multidisciplinary care approach in kidney transplantation, and its feasibility should be ensured in all clinics that perform kidney transplantation.

## Introduction

Enhanced recovery after surgery (ERAS) is a set of methods that provide early recovery with a multimodal approach in the perioperative care pathway.^1^ ERAS protocols are widely used worldwide in major surgery to improve surgical outcomes and require multidisciplinary collaboration. ERAS protocols can improve pain management, earlier mobilization, improved oral nutrition, and shorter hospital stays. These results suggest both enhanced quality of care and reduced costs.^2^ The outlined guidelines include preoperative education, preoperative nutrition optimization, opioid-independent pain control, early mobilization, and early postoperative oral nutrition.^1^ Even so, the reason for its late introduction into renal transplant programs is that it takes time to be sure of its safety and efficacy in these high-risk patients.^3^ ERAS for renal transplant patients was only put on the agenda in 2019 when it was realized that there was extremely poor standardization as a result of the study by Morkane et al.^4^ investigating perioperative renal transplantation practice in 23 different centers. The main points here are demonstrating the importance of ERAS in renal transplantation patients and the management facilities providing ERAS to such patients (Table 1).

### Preoperative Preparation

A good ERAS protocol should start from the surgical preparation phase. Preparing a patient for transplantation can be challenging.

**Patient education and counseling:** All patients should be subjected to a preoperative education process about the operation process and its aftermath, possible complications, and how to deal with them. Thus, the patient will be able to reshape his/her expectations about surgery, be psychologically well prepared, and increase his/her belief in rapid recovery.^5^ When patients are advised to lose weight and quit smoking before surgery, the process will be much more successful. Although the optimal time to quit smoking is not known, Kidney Disease: Improving Global Outcomes states that smoking should be stopped at least 4 weeks before surgery, and the risks will decrease in proportion, and the greater the number of weeks of quit, the lower the risks.^6^ With good counseling, the uneasiness of patients with surgical anxiety can be overcome, and this will make things easier, even with pain control.^5^

**Medical optimization: **If the patient is evaluated well in terms of cardiac, respiratory, and metabolic aspects and is operated on in the best possible condition, the risk of adverse events will decrease. The rate of cardiac disease comorbidity in patients with end-stage renal failure may be 5-30 times higher than that in the normal population.^7^ Patients should be evaluated and treated by a cardiologist who is included in the team and who is familiar with the cardiac management of transplant recipients. Non-invasive coronary tests should be performed in patients deemed necessary, and even the need for percutaneous coronary angiography should be evaluated. Patients with unstable coronary vascular disease, valvular disease, and heart failure should undergo repeated cardiac evaluations because they are on the waiting list and may need to be prepared for emergency surgery.^2^ The optimal screening frequency for cardiac asymptomatic transplant candidates is unknown.Transplant candidates are 2-8 times more likely to be diagnosed with pulmonary hypertension than the normal population because of fistulas used for dialysis, fluid overload, anemia, impaired left ventricular function, and endothelial structure. In particular, patients with a pulmonary pressure >35 mmHg are considered at high risk of morbidity and mortality. The flow and duration of the fistula are also related to the severity of pulmonary hypertension. Pulmonary hypertension should be treated with vasodilator agents in a good preoperative preparation.If patients have substance abuse during preoperative preparation, have heart disease that cardiologists are still treating, have high pulmonary pressures and ongoing problems in lung volumes and capacities, a new stroke, or active peripheral arterial disease; a good ERAS optimization would be to postpone the surgery of these patients until the treatments are completed.^6^

Anemia treatment is also part of medical optimization. It should be remembered that wound infection, pneumonia, sepsis, and mortality are more common in patients who are anemic and require intraoperative blood transfusion. Oral or intravenous iron therapy should be started in the preoperative evaluation of appropriate patients diagnosed with anemia (Hgb≤11 g dL^-1^). Anemia in patients with renal failure should be managed if the hemoglobin (Hb) level falls to 11 g L^-1^ or becomes symptomatic.^2^ Erythropoietin treatment should not be avoided when necessary. Graft life may be shortened in patients with anemia, and the goal should be a normal Hb level after transplantation. Another important point of care for hematological problems is coagulation problems. Dialysis anticoagulation, medical treatments, and intraoperative heparinization should all be considered and discussed in detail with the patient, and central blocks instead of general anaesthesia should be avoided. Hemodialysis the day before transplantation will optimize fluid status, correct electrolyte imbalance, and prevent metabolic imbalance. Because of the risks of sudden and unintended fluid imbalance, dialysis should not be performed on the day of transplantation unless very high potassium levels and fluid overload are present.

**Improving nutritional status and carbohydrate loading: **Intense inflammation, loss of appetite, uremia, long fasting periods before the procedure, and high comorbidities can cause poor nutritional status. Because postoperative morbidity will be higher in patients with malnutrition, nutritional correction should be implemented according to ERAS protocols. Oral nutritional supplements and enteral or parenteral nutrition solutions should be started at least seven days before transplantation. Serum albumin levels should be maintained within the normal range. Kidney transplantation patients are usually placed on preoperative dialysis in preparation for transplantation and may remain dehydrated. Clear liquids should be consumed up to the last two hours on the morning of the operation. Preoperative carbohydrate-rich clear liquid food, which accelerates recovery and shortens the fasting period, is an important step for ERAS. Rapid recovery from the surgical catabolic process can be achieved using carbohydrate-rich fluids.^1^ Preoperative administration of solutions containing complex carbohydrates, such as maltodextrin, is recommended by ERAS associations and the European Society of Anesthesia. The advantages of carbohydrate loading include optimizing metabolism, increasing insulin sensitivity, decreasing nausea and vomiting, and decreasing anxiety.^5^ Non-diabetic kidney recipients may benefit from preoperative carbohydrate loading.^3^ In preoperative transplant recipients who consume carbohydrate beverages, postoperative nausea and vomiting will decrease, insulin resistance will improve, and hospital stay will decrease.^2^

**Anxiolysis:** Anxiety may complicate anaesthesia induction, maintenance, and postoperative recovery. There are no studies in the literature showing a relationship between premedication and graft function. Unfortunately, there is not enough information about the best premedication medication for kidney transplant recipients. However, the necessity of adequate preoperative information and psychological support cannot be ruled out. While premedication is recommended for patients with intense anxiety, adequate psychological support is sufficient for others. Premedication with a proton pump inhibitor or H_2_ inhibitor in patients with slow gastric emptying can reduce the risk of aspiration.

### Intraoperative Care

**Standard Anaesthesia Protocol:** While propofol or thiopental is safe for induction, ketamine should be avoided, especially in patients with ischemic heart disease associated with renal failure. The selection of muscle relaxants and reversal drugs should be appropriate for renal failure. Thus, atracurium or cisatracurium is the most appropriate option.^2^ Opioid derivatives, except morphine, can be safely used intraoperatively, but it may be more beneficial to avoid them in ERAS protocols for postoperative pain control. Sevoflurane or desflurane inhalation anaesthesia or intravenous infusion anaesthesia with propofol may be appropriate. Norepinephrine is a suitable alternative for patients requiring intraoperative vasopressors.^8^ Prevention of nausea and vomiting at the end of surgery is an important pillar of ERAS protocols. Medical treatment includes ondansetron therapy and avoiding high doses of opioids. Nausea and vomiting delay discharge, decrease patient satisfaction, delay early oral feeding, prolong hospital stay and increase costs. These are important points that need to be addressed in ERAS protocols. Immunosuppressive methylprednisolone may prevent nausea and vomiting, but standard nausea and vomiting prophylaxis may also be helpful in ERAS.

**Targeted fluid therapy: **Optimized perioperative hemodynamic management can prevent delayed graft function. Current data support the use of targeted fluid therapy during kidney transplantation. It should be recognized that central venous pressure (CVP) measurement is not a precise and reliable method for assessing fluid status in most cases.^1^ However, comparing the baseline CVP values with the intraoperative ongoing changes may direct fluid therapy; therefore, central venous catheterization is usually recommended for recipients. The use of transesophageal echocardiography, pulse-volume index, and pulse-pressure index monitoring can prevent cardiovascular complications, fluid overload, and delayed graft function.^3^ Fluid overload will increase the weight of the kidney transplant patient, cause fluid overload in the lungs, cause intestinal wall edema, and ultimately, cause prolonged ileus and delayed discharge. As a result, the best fluid resuscitation principle should be: “As much as needed, as little as possible”.^9^ The proper fluid type remains controversial and open for research, but balanced crystalloid solutions seem to be the best choice.^1, 9^ A goal-directed fluid therapy is performed with crystalloid infusion at 3-5 mL kg^-1^ h^-1^ supplemented with 5% albumin if needed.^1^ Diuresis induced by diuretic agents is recommended, especially immediately after reperfusion, and is particularly important for verifying successful transplantation.

**Perioperative hyperglycemia control:** Diagnosis of diabetes and the type of steroid used in treatment can complicate glycaemic control. All patients with pre-diabetes and 66% of transplant recipients without a diagnosis of diabetes require insulin treatment after transplantation.^10^ In the ERAS application recommendations, blood glucose should be targeted at 140-189 mg dL^-1^ during the perioperative period.^11^

**Temperature control:** Hypothermia can cause perioperative complications, such as wound infection, coagulation disorders, increased transfusion requirements, impaired drug metabolism, and delayed awakening. Each of these would be sufficient to disrupt the proper progression of the ERAS protocol. Patient warming should have started from the ward to the operating room. The temperature should be recorded before sedation and measured every 30 minutes. The central temperature should be targeted at 36.0 ºC and above, and the patient should be warmed throughout the surgery. The warming process must be continued in the recovery room.^12^

### Postoperative Management

**Early mobilization: **Poor physical activity is associated with poor quality of life. Early mobilization can reduce both the complication rates and duration of hospital stay. Respiratory and thromboembolic complications associated with prolonged bed rest can be easily prevented with early mobilization.^5^ Lack of physical activity may also trigger muscle atrophy, joint movement limitation, pressure ulcers, and atelectasis.^13^ Patients’ quality of life will improve with mobilization. Exercises started in the early period will increase functional capacity, muscle strength, and quality of life.

**Early enteral nutrition:** According to traditional wisdom, the initiation of postoperative oral nutrition was slow and gradual. In this practice, transparent liquid foods were introduced, followed by a gradual transition to solid foods. However, new evidence suggests that feeding should be started as early as possible after surgery, either orally or through a nasogastric tube. There is no need for a routine nasogastric tube. Patients can tolerate oral fluids quickly. Moreover, they can be rapidly switched to a routine postoperative diet.

**Catheter and drain maintenance: **Leaving a foley catheter in place for a prolonged period is a high-risk factor for urinary tract infection. The early removal of postoperative catheters is safe for a good ERAS protocol. If a patient has a prophylactic J-stent, it should be removed as early as possible to reduce the risk of postoperative infection and stenosis.

**Pain control: **In addition to reducing postoperative pain, multimodal pain control in the ERAS guidelines is necessary to facilitate early oral food intake, early mobilization, and accelerated surgical recovery.^14^ The effects of opioid use in postoperative pain control, such as slowing down bowel movements, and side effects, such as dizziness, nausea, vomiting, blurred vision, late mobilization, disruption of the mucosal immune response of host defense mechanisms, and disruption of the microbiota leading to infection of some pathogens, have led to opioid-independent pain control in ERAS steps.^1^ Furthermore, unlike ERAS protocols used in other surgeries, nonsteroidal anti-inflammatory drugs have no place in pain control in renal transplantation ERAS protocol because of their nephrotoxicity. Therefore, local anaesthetics, surgical incision infiltration anaesthesia, and plane blocks are the building blocks of the multimodal approach to renal transplant ERAS pain control.^1^ Performing a plane block reduces the need for opioids and the associated incidence of nausea and vomiting, prevents slowing of bowel movements, and facilitates early mobilization.^2^

In conclusion, even though ERAS protocols have entered practice later than other surgeries, it is possible to implement ERAS protocols in renal transplantation by adapting them to the specific dynamics of the transplantation process. The ERAS program should be considered a multidisciplinary care approach in kidney transplantation, and its feasibility should be ensured in all clinics that perform kidney transplantation.

## Figures and Tables

**Table 1. ERAS Protocol for Renal Transplant Patients15:** 

**Preoperative**	**Perioperative**	**Post-operative**
Oral and written information and patient education	Carbohydrate loading and oral fluid storage until 4 h before surgery	Goal-directed fluid therapy
Smoking cessation	TED stockings	Early mobilization
Avarage weight and blood pressure	Anxiolysis	Early oral feeding
Cardiac optimization	Proper induction and maintenance anaesthesia agents	Early removal of drains and catheter
Anemia management	Goal-directed fluid therapy using crystalloids	Education about drugs and doses and the outpatient review protocol
-	-	Post-discharge outpatient clinic review

ERAS, Enhanced Recovery After Surgery; TED, thromboembolism deterrent.
